# Biofilm formation by *Mycobacterium avium *isolates originating from humans, swine and birds

**DOI:** 10.1186/1471-2180-9-159

**Published:** 2009-08-06

**Authors:** Tone Bjordal Johansen, Angelika Agdestein, Ingrid Olsen, Sigrun Fredsvold Nilsen, Gudmund Holstad, Berit Djønne

**Affiliations:** 1 Department of Animal Health, National Veterinary Institute, Pb.750 Sentrum, 0106 Oslo, Norway; 2 Norwegian School of Veterinary Science, PO Box 8156 Dep, 0033 Oslo, Norway

## Abstract

**Background:**

*Mycobacterium avium *includes the subspecies *avium*, *silvaticum*, *paratuberculosis *and *hominissuis*, and *M. avium *subspecies has been isolated from various environments all over the world including from biofilms in water distribution systems. The aim of this study was to examine isolates of *M. avium *subsp. *avium *and *M. avium *subsp. *hominissuis *of different origin for biofilm formation and to look for correlations between biofilm formation and RFLP-types, and to standardise the method to test for biofilm formation. In order to determine the best screening method, a panel of 14 isolates of *M. avium *subsp. *avium *and *M. avium *subsp. *hominissuis*, were tested for their ability to form biofilm in microtiter plates under different conditions. Subsequently, 83 additional isolates from humans, swine and birds were tested for biofilm formation. The isolates were tested for the presence of selected genes involved in the synthesis of glycopeptidolipids (GPLs) in the cell wall of *M. avium*, which is believed to be important for biofilm formation. Colony morphology and *hsp65 *sequvar were also determined.

**Results:**

Nine isolates from swine produced biofilm. There was a significant higher frequency of porcine isolates forming biofilm compared to human isolates. All isolates were previously characterised by IS*1311*- and IS*1245*-RFLP typing. The ability to form biofilm did not correlate with the RFLP-type, *hsp65 *sequevar, colony morphology or the presence of gene sequences related to GPL synthesis.

**Conclusion:**

The observed differences in biofilm forming abilities between porcine and human isolates raises questions regarding the importance of biofilm formation for infectious potential. The optimised method worked well for screening of multiple isolates.

## Background

*Mycobacterium avium *includes the subspecies *avium*, *silvaticum*, *paratuberculosis *and *hominissuis *[1-3]. The former, *M. avium *subsp. *avium *causes tuberculosis in captive and free living birds [4], while *M. avium *subsp. *hominissuis *is an opportunistic environmental pathogen for humans and swine, and occasionally also for other mammals [1]. The most common forms of disease in humans are pulmonary disease, lymphadenitis and disseminated infection [5-7], while swine usually develop localised lymph node lesions [8]. Various molecular tools have been used to characterise isolates of *M. avium*, including restriction fragment length polymorphism (RFLP) [9], sequencing of the *hsp65 *gene [10] and multilocus sequence analysis (MLSA) [11]. In a previous study, we characterised *M. avium *isolates from birds, swine and humans in Norway by IS*1311*- and IS*1245*-RFLP typing. Our study demonstrated that transmission between animals and/or humans of identical isolates of *M. avium *is uncommon in Norway, and that transmission of *M. avium *from the environment to humans and animals is more likely [12]. The results are in accordance with other studies [13-15].

*M. avium *has been found in soils and waters worldwide [5], and isolates with identical RFLP-profiles have been found in peat and human patients and in peat and swine, respectively [16,17]. Drinking water has also been shown to be a possible source of *M. avium *subsp. *hominissuis *for both humans and swine [18-21]. *M. avium *has been shown to survive in water for up to 26 months, and can also survive within amoeba [22,23]. Additionally, potable hot water systems may contain *M. avium *concentrations greater than those found in cold water systems [24]. In natural settings, bacteria on surfaces and interfaces are found as multicellular aggregates, called biofilms [25]. *M. avium *has been detected in naturally occurring biofilms in water distribution systems, and has been shown to persist in drinking water biofilms for weeks [20,26]. *M. avium *may survive traditional water disinfection procedures because it is naturally resistant to water treatment with ozone and chlorine, and has been shown to be even more resistant to chlorine treatment when grown in biofilm [22,27,28]. Biofilms in drinking water systems may, therefore, be of importance as a reservoir for *M. avium*, and bacteria could be transmitted to humans and animals with drinking water. Biofilm formation in *M. avium *has been evaluated in vitro, and the ability to form biofilm varies between isolates and under different growth conditions [29,30]. So far, biofilm studies of *M. avium *have been performed with only a few human and environmental isolates, and biofilm studies of isolates from birds and swine have, to the authors' knowledge, not been reported.

Glycopeptidolipids (GPLs), present in the outermost layer of the cell wall of *M. avium *and *M. smegmatis*, seem to be of importance for biofilm formation in both species [29,31-33]. The GPLs of *M. avium *can be divided into non-serovar-specific (nsGPL) and serovars-specific GPL (ssGPL) [34]. Whether different serovars have different abilities to make GPL, is not known. Furthermore, GPLs are associated with colony morphology, and *M. avium *colonies can be smooth opaque (SmO), smooth transparent (SmT) or rough (Rg) [35,36]. The Rg variants of *M. avium *have been shown to have alterations in their GPLs [37].

The aim of the present study was to screen a large number of *M. avium *isolates of different origin for biofilm formation, and to correlate the ability to form biofilm with RFLP-types (published previously [12]) and *hsp65 *sequevars of the isolates. In addition, we wanted to examine the presence of some selected genes sequences in the GPL biosynthesis gene cluster to elucidate the importance of GPLs for biofilm formation and colony morphology. To do this, the biofilm screening method needed optimisation.

## Methods

**Figure 1:**
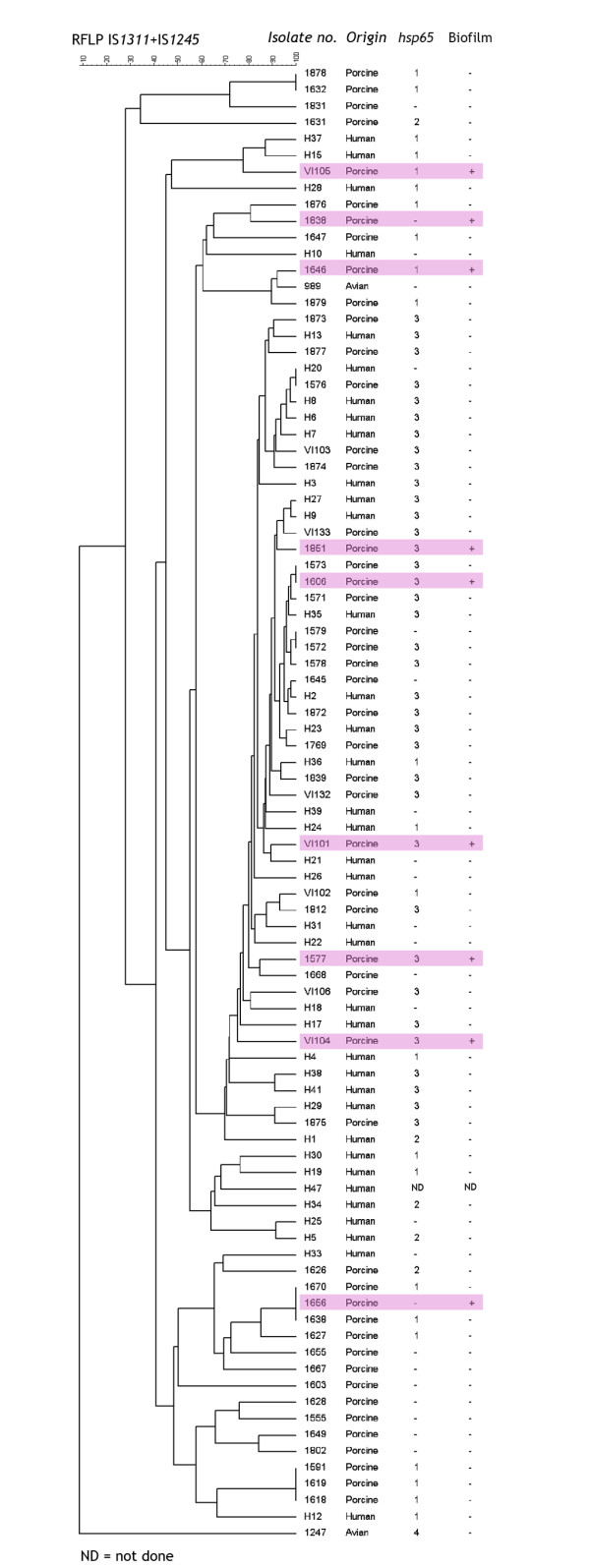
**Distribution of biofilm producing *Mycobacterium avium* isolates in a dendrogram based on the cluster analysis of the composite dataset of RFLP typing using both IS*1311* and IS*1245* as probes**. A total of nine isolates of *M. avium *subspecies *avium *and 88 isolates of *M. avium *subsp. *hominissuis *isolated in Norway were included. The RFLP dendrogram has been presented elsewhere [12], but is has presently been combined with additional information regarding *hsp65 *code and the biofilm forming abilities of the isolates. #1247 represents the identical profiles of nine avian isolates, including #1553 and #1794. Biofilm forming isolates have been highlighted in pink.

Eighty-eight Norwegian isolates of *M. avium *subspecies *hominissuis *from human patients (n = 36), swine (n = 51) and one bird and nine isolates *M. avium *subspecies *avium *originating from wild birds were examined for their ability to form biofilm (Figure 1). The isolates have been described previously [12]. In addition, the reference strains *M. avium *ATCC 25291, R13 and *M. avium *104 were examined. *M. smegmatis *mc^2 ^was included as a positive control for biofilm formation.

### Method optimisation

A panel of 14 *M. avium *subsp. *hominissuis *(seven from humans, six from swine and one from a bird), and three *M. avium *subsp. *avium *isolates originating from birds, including the reference strains ATCC 25291 and R13, and the positive control *M. smegmatis *mc^2 ^were used during optimisation of the method. The isolates all had a low passage number.

Biofilm formation was determined as previously described [30], but with some modifications. Isolates were cultured in 10 ml Middlebrook 7H9 (BD Diagnostics, Sparks, MD) containing 10% oleic acid, albumin, dextrose and catalase (BD Diagnostics), 0.1% Tween 80 (Merck KGaA, Darmstadt, Germany) and 0.2% glycerin (Merck) (7H9 with OADC and Tween). They were incubated with agitation (100/min) at 37°C for minimum two weeks until they reached the stationary phase at which point culture aliquots were frozen at -70°C. Subsequently, 100 μl of frozen stock culture was inoculated in 10 ml of fresh 7H9 with OADC and Tween and incubated at 37°C with agitation for seven days. OD_600 _was measured, and the cultures were centrifuged and resuspended to an OD_600 _of 0.2 in the different medias described below. 200 μl of the cell suspension were added to the wells of a 96-well flat bottom polystyrene microtiter plate in triplicates (MicroWell™ Plates Nunclon™Δ no. 167008 (Nunc, Nuncleon, Roskilde, Denmark) [38], and incubated without agitation in a sealed container with 20 ml sterile distilled water to prevent drying. Medium without bacteria were used as negative controls on each plate.

After incubation for two or three weeks, bacterial growth was determined by OD_595 _measurement. The wells were washed once with 250 μl tap water, and the remaining biofilm was stained using 250 μl 1% crystal violet (Sigma-Aldrich, St. Luis, MO), followed by 30 minutes incubation at room temperature. The wells were rinsed three or four times with tap water to remove unbound dye before the stained biofilm was resuspended in 250 μl ethanol: acetone 70:30. Finally, the amount of biofilm was measured at OD_595_. Results were presented as the median value of the triplicates, subtracting the median value for the negative control.

The different media examined were: Middlebrook 7H9 with OADC and Tween, Middlebrook 7H9 without OADC and Tween, a mixture of 50% sterile distilled water and 50% Middlebrook 7H9 with OADC and Tween, sterile Hanks' Balanced Salt solution (Sigma-Aldrich), distilled water and sterile filtrated or autoclaved tap water and lake water. Different temperatures; 37°C, 28°C and 20°C, and incubation time; two and three weeks, were tested using Middlebrook 7H9 with OADC and Tween.

### Screening of isolates

Based on the results from the method optimisation, Middlebrook 7H9 with OADC and Tween, and incubation for two weeks at 20°C was selected to screen the 97 isolates, and the reference strains R13, ATCC25291 and *M. avium *104 for biofilm formation. Positive control, *M. smegmatis *mc^2 ^and negative control, Middlebrook 7H9 with OADC and Tween, were included on each plate. All samples were examined in triplicates. The amount of biofilm was determined as described above, with a slight modification. Before staining, 250 μl methanol was used to wash the wells before the plate was left to dry for 15 min. This methanol fixation gave less variability between repeated assays. Biofilm was stained with crystal violet as described above.

### Sequencing of *hsp65*

The *hsp65 *sequencing was performed as described by Turenne et al [10]. Briefly, a 1059 bp fragment of the *hsp65 *gene was amplified by PCR, and the product was sequenced and analysed by BioEdit (Ibis Biosciences, Carlsbad, CA). Isolates were assigned to *hsp65 *codes based on the presence of single nucleotide polymorphisms (SNPs) compared to the reference strain *M. avium *104.

### Colony morphology

The colony morphology of all isolates was examined on Middelbrook 7H10 (BD Diagnostics) medium after incubation at 37°C for two, three, four and five weeks. Colonies were described as smooth transparent (SmT), smoth opaque (SmO) or rough (Rg) [35].

### GPL biosynthesis genes

Primers for the GPL biosynthesis genes *mdhtA*, *merA*, *mtfF *(called *gsc *by [39]), *rtfA*, *mtfC *and *gtfA *[39,40] were designed using the programme Primer 3 http://frodo.wi.mit.edu/primer3/. Primers and Genbank accession numbers for the various genes are listed in Table 1. PCR reactions were performed with Taq DNA polymerase (Qiagen, Hilden, Germany) with the following PCR program: Initial denaturation at 94°C for 3 min, followed by 30 cycles of 94°C for 30 sec, 58°C for 30 sec and 72°C for 30 sec. PCR products were resolved by gel electrophoresis, stained with ethidium bromide and visualised and captured under UV-light. All nine biofilm forming isolates and nine isolates closely related to these based on RFLP results [12], ten isolates harbouring IS*Mpa1 *[12,41] and 13 other isolates were screened for the presence of the six GPL biosynthesis genes. All together 42 isolates were examined (27 isolates from swine, ten from humans and five from birds including the reference strains ATCC 25291, R13 and *M. avium *104).

**Table 1:** Primers and GenBank coding positions for the glycopeptidolipid (GPL) genes examined in this study

Gene	AF125999** coding position**	Primer sequence	Start-stop within gene (prod size in bp)
*merA*	15360–16379	P102 tattgactggccctttggag	452–659 (208)
		P103 gctttggcttcctcatatcg	
*mtfF*	16655–17377	P104 gctgccgatgcttaaaagtc	342–499 (158)
		P105 gcttctcgaaaccctgtacg	
*mdhtA*	14389–15420	P106 gacccggatgaggtctacaa	232–402 (171)
		P107 gaacatctccgacgaggaag	
*rtfA*	4488–5774	P108 ccattggtcgtgaactgatg	56–214 (159)
		P109 ttttgaagaagtcccggatg	
*gtfA*	2807–4084	P112 ttctggaagatgggggagat	223–400 (178)
		P113 gcggaaggtcgtaatactcg	
*mtfC*	5876–6676	P114 ggcgtgatctgaccaggtat	44–266 (223)
		P115 tcttccagaaccgtttccac	

## Results

### Method optimisation

**Figure 2:**
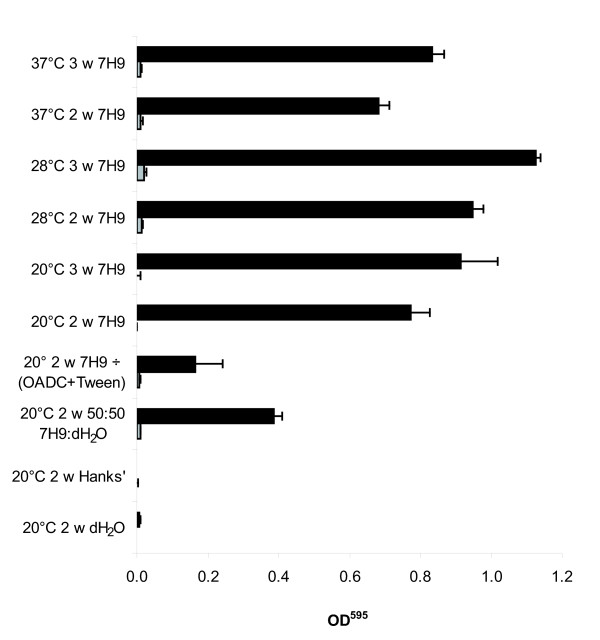
**Biofilm formation for the different conditions tested**. Fourteen *Mycobacterium avium* subspecies *hominissuis* (seven from humans, six from swine, one from a bird), and three *M. avium *subsp.*avium *isolates from birds were used to optimise the method. Results are represented as mean OD^595 ^value after crystal violet staining of biofilm + SEM (Standard error of the mean). Isolates forming biofilm (#1646, #1838, #1851, #VI101) are illustrated with black bars, and isolates not forming (#H1, #H3, #H5, #H12, #H15, #H28, #H38, #1591, #1831, #989, #1247, #ATTC25291, #R13) as grey bars. Abbreviations; w = week; 7H9 = Middlebrook 7H9 with OADC and Tween; 7H9 ÷ (OADC+Tween) = Middlebrook 7H9 with neither OADC nor Tween; 50:50 7H9:dH_2_O = 50% Middlebrook 7H9 with OADC and Tween and 50% distilled water; Hanks' = Hanks' balanced salt solution and dH_2_O = distilled water.

Biofilm formation by the 17 isolates of *M. avium *with respect to incubation time, temperature and media is described in Figure 2. Only four isolates formed biofilm, and the greatest amount of biofilm was obtained using 7H9 with OADC and Tween. A mixture of 50% sterile distilled water and 50% 7H9 with OADC and Tween or 7H9 without OADC and Tween both gave less biofilm formation. None of the isolates showed growth or formed biofilm when incubated in Hanks' balanced salt solution or water from different sources, including distilled water, sterile filtrated or autoclaved potable water and lake water (results not shown). All temperatures and incubation times tested gave good biofilm formation by the biofilm positive isolates using 7H9 with OADC and Tween as medium. The best results were obtained at 28°C and by using three weeks of incubation. The trait of biofilm production was consistent between the isolates, and the non-biofilm forming isolates were negative under all conditions (Figure 2).

### Screening of isolates

**Figure 3:**
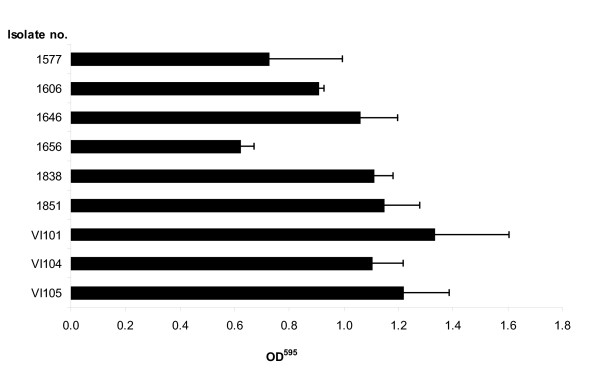
**Differences in the amount of biofilm formed in microtiterplates amongst the nine isolates forming biofilm**. Results are represented as mean OD^595 ^value after crystal violet staining of biofilm+ SEM. The calculations of mean values are based on triplicates repeated two to three times. The nine isolates were all of porcine origin.

Based on the results from the method optimisation, all 97 isolates plus reference strains were screened using 7H9 medium with OADC and Tween. For practical reasons and in order to mimic environmental conditions, incubation at 20°C (room temperature) for two weeks was chosen. Nine of the 97 isolates formed biofilm; all were of porcine origin and had average OD_595 _values ranging from 0.62 to 1.22 (Figure 3). The remaining isolates had OD_595 _values below 0.10 and were not regarded as biofilm forming isolates. Neither the ten bird isolates nor the 36 human isolates formed biofilm. The difference in biofilm forming abilities of isolates from swine as opposed to isolates from humans was significant by the Fisher Exact Test (p < 0.05). Isolates that formed biofilm belonged to nine different RFLP profiles (Figure 1), and were not genetically related based on RFLP typing.

### Sequencing of *hsp65 *and colony morphology

Sequencing of the *hsp65 *gene to detect single nucleotide polymorphisms (SNPs) was selected as a second method to distinguish between isolates of *M. avium*. The method was chosen as a complementary analysis in addition to RFLP, because it targets a genetic element that is more stable than the IS elements, with a slower "molecular clock". Seventy-two isolates were sequenced to determine the *hsp65 *code, and the results are presented in Figure 1 and Table 2. All the bird isolates (*M. avium *subsp. *avium*) belonged to *hsp65 *code 4, and the human and porcine isolates (*M. avium *subsp. *hominissuis*) belonged to *hsp65 *codes 1, 2 and 3. The biofilm forming isolates from swine were either code 1 or code 3, but no correlation between *hsp65 *code and ability to form biofilm could be detected.

**Table 2:** *Hsp65 *code amongst the 72 tested *Mycobacterium avium *isolates of different origin.

	*hsp65 *code				
**Origin**	**1**	**2**	**3**	**4**	

**Avian**				8 (100%)	8 (100%)
**Human**	9 (34%)	3 (12%)	14 (54%)		26 (100%)
**Biofilm forming porcine**	2 (29%)		5 (71%)		7 (100%)
**Biofilm non-forming porcine**	12 (39%)	2 (6%)	17 (55%)		31 (100%)
**Total**	23 (32%)	5 (7%)	36 (50%)	8 (11%)	72 (100%)

Ref. strains are not included in the table.

All isolates, except one, were either SmT or SmO after two weeks of incubation (Table 3). The reference strain ATCC 25291 was the only Rg isolate after two weeks. A few isolates had a combination of SmT and SmO colonies, and one isolate (#1667) displayed a combination of SmO and Rg colonies. After three, four and five weeks of incubation the morphology changed for many of the isolates. The results are in accordance with other studies [37]. Amongst the biofilm forming isolates, both SmT and SmO colonies were observed, but none of these isolates had Rg colony morphology after two weeks.

**Table 3:** Colony morphology observed after two weeks incubation on Middlebrook 7H10 agar at 37°C.

Colony morphology				
**Origin**	**SmT^1^**	**SmO^2^**	**Intermediate**	**Total**

**Avian**	8 (80%)	2 (20%)		10 (100%)
**Human**	15 (42%)	18 (50%)	3 (8%)	36 (100%)
**Biofilm forming porcine**	7 (78%)	2 (22%)		9 (100%)
**Biofilm non-forming porcine**	19 (45%)	20 (48%)	3 (7%)	42 (100%)
**Total**	49 (51%)	42 (43%)	6 (6%)	97 (100%)

^1^Smooth transparent

^2^Smooth opaque

The reference strain ATCC 25291 was the only rough (Rg) isolate after two weeks.

Ref. strains are not included in the table.

### GPL biosynthesis genes

The isolates were divided into three groups based on PCR detection of the six genes (Table 4). Group I (14 isolates) were positive for all genes examined (*gtfA*, r*tfA*, *mtfC*, *mdhtA*, *merA *and *mtfF*). Four biofilm forming isolates and all five isolates from birds (four *M. avium *subsp. *avium *and one *M. avium *subsp. *hominissuis*), including the two reference strains, belonged to this group. Group II consisted of 18 isolates negative for the *ser2 *cluster genes *mdhtA*, *merA *and *mtfF *and positive for the nsGPL genes *gtfA, rtfA *and *mtfC*. Four biofilm forming isolates belonged to this group. One isolate from swine in this group harboured IS*Mpa1 *[41]. Group III (nine isolates) were negative for all genes tested. All of these isolates harboured the IS*Mpa1*- element [12,41], and one of them (#1656) formed biofilm. Two isolates (#1591 and # 1655) had weak positive reactions to the *mtfC*-PCR. Sequencing showed that they had a few basepair differences compared to AF125999/TMC724 (ATCC 25291). The PCR product of #1591 was identical to the *mtfC *sequence of *M. avium *104. In the pairs of isolates with similar or identical RFLP profiles where one formed biofilm and the other did not, five pairs had the same profile of genes, while three pairs did not. The presence or absence of these genes did not correlate with biofilm formation, as biofilm forming isolates were present in all three groups.

**Table 4:** Presence of genes related to glycopeptidolipid synthesis, biofilm-formation, RFLP-clustering, presence of IS*Mpa1 *and *hsp65*-code among *Mycobacterium avium *isolates.

Isolates	Origin	Relation^1^	IS*Mpa1*	*hsp65*	nsGPL genes^2^	*ser2 *genes^3^
**Group I**						
989	Bird		-	-	+	+
1553,1794	Bird		-	4	+	+
ATCC 25291	Ref str.		-	-	+	+
R13	Ref str.		-	4	+	+
H31	Human		-	-	+	+
H38	Human		-	3	+	+
**VI105**	Swine	H15	-	1	+	+
1572, 1573, VI133	Swine		-	3	+	+
**1577**	Swine	1668	-	3	+	+
**1606**	Swine	1573	-	3	+	+
**1851**	Swine	VI133	-	3	+	+

**Group II**						
H21, H22	Human		-	-	+	-
H12, H15, H28	Human		-	1	+	-
H1	Human		-	2	+	-
H17	Human		-	3	+	-
1555, 1628	Swine		-	-	+	-
1603	Swine		+	-	+	-
1668, 1831	Swine		-	-	+	-
**1838**	Swine	1876	-	-	+	-
**1646**	Swine	989	-	1	+	-
1876, 1878	Swine		-	1	+	-
**VI101**	Swine	H21	-	3	+	-
**VI104**	Swine	H17	-	3	+	-

**Group III**						
1655, 1591	Swine		+	-	-^4^	-
1649, 1802	Swine		+	-	-	-
**1656**	Swine	1670	+	-	-	-
1627, 1638, 1670	Swine		+	1	-	-

The isolates are divided into three groups, I, II and III, based on presence or absence of GPL genes.

^1^Isolate related by RFLP (Figure 1)

^2^nsGPL genes: *gtfA*, *rtfA *and *mtfC*

^3^*ser2 *genes: *mdhtA*, *merA *and *mtfF*

^4^1591 and 1655 had a weak PCR product for *mtfC*. Sequencing showed a product with few bases different from AF125999 TMC724/ATCC 25291). The PCR product of #1591 was identical to the sequence of the *mtfC *gene of *M. avium *104

Biofilm forming isolates are marked in bold typing.

## Discussion

In this study, a method suitable for screening a large number of *M. avium *isolates for biofilm formation was established. Ninety-seven isolates of *M. avium *subsp. *avium *and *M. avium *subsp. *hominissuis *originating from birds, swine and humans were examined for their biofilm forming abilities. To our knowledge, this is the first time a large number of such isolates from different hosts have been tested for biofilm formation. Nine isolates from swine formed biofilm, none of the isolates from humans or birds did. The optimised method was easy to perform, can be adapted to other test-conditions and gave clear and consistent results. A high and consistent biofilm-production was seen only when using Middlebrook 7H9, while no biofilm was detected in water. Biofilm forming abilities did not correlate with RFLP-profile, *hsp65 *sequevar, colony morphology or with the presence of the tested GPL biosynthesis genes.

Water has been described as the best medium for evaluation of biofilm formation [30,42]. Williams et al used autoclaved potable water for biofilm quantification by CFU count and imaging [42], while Geier et al. used MQ water [43]. However, our isolates did not make biofilm in water, even though different types of water and water from different sources like distilled, potable and lake water was included. This discrepancy between earlier studies and the present study can be due to different isolates tested or to other conditions in the experimental set-up. Water is not a standardised medium, and the content of ions, organic matter and the pH will vary depending on local factors. Carter et al. demonstrated the effect of different ions and carbon sources on biofilm formation [30]. To test a medium containing different salts and glucose, we tested our panel of isolates in Hanks's balanced salt solution, which has been described as potential biofilm media for *M. avium *[33,42]. However in our hands, none of the isolates formed biofilm in Hanks'.

In the present study, few isolates formed biofilm. The testing is performed under laboratory conditions, and cannot be directly transferred to bacterial behaviour in the environment. Under natural conditions bacteria are part of multispecies communities, and in nature it is possible that any *M. avium *isolates can be found in biofilm, regardless of whether or not it shows the ability for biofilm production under laboratory conditions. To form a biofilm, planctonic bacteria must first attach to a surface. Thereafter, they can organise into a biofilm, first as microcolonies then as macrocolonies [44]. This organising of bacterial cells is regulated by intraspecies and interspecies cell communication [45]. The autoinducer AI-2 is a universal quorum sensing signal used by many bacteria for interspecies communication [45]. *M. avium *has been shown to increase biofilm formation in response to AI-2, and to culture supernatant from a good biofilm producer [30,43]. We tested the ability to form biofilm in the laboratory under given conditions, and under such conditions, bacteria may not form biofilm due to the absence of stimuli from a microbial community.

Results from typing using IS*1245*- and IS*1311*-RFLP profiles and *hsp65*-sequevar did not correlate with the ability to form biofilm. Even apparently genetically similar isolates, like # 1606 and # 1573 that had identical RFLP profiles, belonged to the same *hsp65 *sequevar and showed identical results by PCRs for the GPL genes, had different ability to form biofilm. Biofilm formation is probably a complex process controlled by many different gene mechanisms. The RFLP method and other fingerprinting methods are suitable for epidemiological surveys and outbreak investigations [46,47], while sequencing of the *hsp65 *gene can be used to phylogenetic studies [48]. In the study of complex mechanisms like biofilm and virulence, the correlation with these typing methods seemed limited.

It has been stated that GPLs are necessary for *M. smegmatis *to form biofilm, and that GPL-deficient mutants do not produce biofilm [31]. Similar findings are reported for *M. avium *[29,33]. In a study performed by Krzywinska and Schorey, the authors found differences between *M. avium *strain A5 and strain 104 regarding the GPL biosynthesis cluster. Strain 104 (serovar 1) lacks several genes belonging to the *ser2 *cluster (serovar 2) [39,40,49], while the genes involved in synthesis of nsGPL are highly conserved [39]. The biofilm producing abilities of these two strains has been described in other studies, and strain 104 produced less biofilm than A5 [30,33]. To investigate the significance of genes in the GPL biosynthesis *ser2 *cluster for the ability to form biofilm, the isolates were screened for the presence of genes involved in the synthesis and modification of nsGPL, serovar 1 and serovar 2 [40,50,51]. The isolates had three different patterns of GPL genes. Strains with a similar organisation as *M. avium *104 and A5 were detected, but there was no association with biofilm formation. In addition one biofilm forming isolate lacked the genes involved in the production of nsGPL. This isolate has previously been serotyped at our institute to be serotype 10. It has been reported that serovars 5 and 10/11 strains do not have the single Rha residue attached by RtfA to 6-dTal, in contrast to all other serovars of *M. avium *[34,52]. The GPL produced by this serotype is not well characterised, but the presented results indicate that they may be able to produce biofilm despite the apparent lack of some genes involved in production of the most common nsGPL. As stated above, GPL has been associated with biofilm forming abilities. In the present study, presence of the GPL genes tested was not correlated with biofilm formation, but an association might be due to expression and not presence of the genes.

The significant differences in biofilm forming abilities observed between porcine and human isolates are surprising since these isolates were very similar when tested for other characteristics. Other studies have reported that isolates of human origin may form biofilm [30,33], so although a significant difference in biofilm formation was observed between human and porcine isolates of *M. avium *subsp. *hominissuis *in the present study, this is not a consistent difference. The ability to invade bronchial epithelial cells has been demonstrated to be impaired in biofilm deficient mutants of the *M. avium *strain A5, and the same mutants had an impaired ability to cause infection in mice [53]. It has thus been suggested that the ability of an isolate to form biofilm is linked to virulence. Biofilm forming isolates may also reach their hosts in large numbers if loosening in clusters from a naturally occurring biofilm. The condition of the host may differ between humans and swine. Human hosts are often immunocompromised or have predisposing lung conditions [6,54], while porcine hosts probably are not. Swine rarely present with clinical disease caused by *M. avium *subsp.*hominissuis *[4]. It could be speculated that swine get infected only when exposed to a large infective dose of the bacterium, for instance originating from naturally occurring biofilms, or that these biofilm related isolates are more virulent. This may lead to a selection for biofilm forming isolates in swine, explaining the differences observed in the present study.

## Conclusion

An optimised method to screen isolates of *Mycobacterium avium *for biofilm formation was established, and this method was used to examine 97 isolates retrieved from humans, swine and birds. Nine isolates, all of porcine origin, formed biofilm. No correlation was found between the ability of the isolates to form biofilm with the presence of selected GPL genes. The biofilm forming isolates were not related by RFLP or *hsp65 *sequencing. The differences observed between the porcine and human isolates raises questions regarding their biofilm forming abilities and the importance of biofilm production for their infectious potential.

## Authors' contributions

TBJ contributed to conception and design, laboratory experiments, analysed data and drafted the manuscript. AA contributed to design, laboratory experiments, analysed data, and the writing of manuscript. SFN contributed to laboratory experiments, data analysis and writing of manuscript. IO, GH and BD contributed to conception and design, data analysis and the writing of manuscript. All authors have read and approved the final manuscript.
